# “Gallstone Hip” and Other Sequelae of Retained Gallstones.

**DOI:** 10.1155/1997/14698

**Published:** 1997

**Authors:** Peter T. Chin, Stuart Boland, John P. Percy

**Affiliations:** 1 Royal North Shore hospital 20/454 Edgecliff Rd Edgecliff New South Wales 2027 Australia; 2 Monavale hospital. 7 Bungan Street Monavale New South Wales 2103 Australia; 3 Royal North Shore hospital. 1A Berry Road St. Leonards New South Wales 2065 Australia

## Abstract

The fate of gallstones spilled during laparoscopic cholecystostomy has been thought to be relatively benign. Recent experience and a review of the recent literature shows that this is not always the case. We report three cases of complications of retained stones and analyse the literature with regard to types of complications, time to presentation, and recommendations for managing spilled gallstones. Retained gallstones have been shown to cause adhesions in the rat and inflammatory reactions in dogs with no evidence of absorption. The average time to presentation of complications arising from retained gallstones is 27.3 weeks. Complications include: Intraabdominal abscess formation with or without abdominal wall sinus tract formation, persisting abdominal wall sinus tracts from port site abscess, subhepatic inflammatory masses, cholelithoptysis, microabscesses and granuloma formation, liver abscess and “dumbell” shaped abscess with one side of the “dumbell” forming a subcutaneous abscess. We recommend the judicious use of retrieval devices during the extraction phase of the laparoscopic cholecystectomy, diligent removal of any spilled stones and awareness of delayed postoperative pain and tenderness as a harbinger of symptomatic retained gallstones. Documentation of intraoperative gallstone spillage, volume, type of gallstones, and effort to retrieve is recommended.


*HPB Surgery*, 1997, Vol.10, pp. 165–168
Reprints available directly from the publisher
Photocopying permitted by license only

© 1997 OPA (Overseas Publishers Association)
Amsterdam B.V. Published in The Netherlands
by Harwood Academic Publishers
Printed in India

### CASE REPORT "Gallstone Hip" and Other Sequelae of Retained Gallstones.

PETER T. CHIN[1], STUART BOLAND[2], JOHN P. PERCY[3]

[1]Royal North Shore hospital. 20/454 Edgecliff Rd, Edgecliff. New South Wales. 2027. Australia.
[2]Monavale hospital. 7 Bungan Street, Monavale. New South Wales. 2103 Australia.
[3]Royal North Shore hospital. 1A Berry Road, St. Leonards, New South Wales. 2065 Australia.

*(Received 1 July 1995)*

The fate of gallstones spilled during laparoscopic cholecystostomy has been thought to be relatively benign. Recent experience and a review of the recent literature shows that this is not always the case. We report three cases of complications of retained stones and analyse the literature with regard to types of complications, time to presentation, and recommendations for managing spilled gallstones. Retained gallstones have been shown to cause adhesions in the rat and inflammatory reactions in dogs with no evidence of absorption. The average time to presentation of complications arising from retained gallstones is 27.3 weeks. Complications include: Intraabdominal abscess formation with or without abdominal wall sinus tract formation, persisting abdominal wall sinus tracts from port site abscess, subhepatic inflammatory masses, cholelithoptysis, microabscesses and granuloma formation, liver abscess and "dumbell" shaped abscess with one side of the "dumbell" forming a subcutaneous abscess. We recommend the judicious use of retrieval devices during the extraction phase of the laparoscopic cholecystectomy, diligent removal of any spilled stones and awareness of delayed postoperative pain and tenderness as a harbinger of symptomatic retained gallstones. Documentation of intraoperative gallstone spillage, volume, type of gallstones, and effort to retrieve is recommended.

KEYWORDS: Gallstones spilled retained laparoscopic cholecystectomy complications gallstone hip abscess cholelithiasis

### INTRODUCTION

Laparoscopic cholecystectomy is now a routine therapy in the management of symptomatic cholelithiasis. As increasing numbers of laparoscopic cholecystectomies are performed, complications specific to this operation are becoming evident. We report three sequelae of spilled gallstones and review the recent literature.

### CASE 1

An 82 year old woman with symptomatic cholelithiasis underwent laparoscopic cholecystectomy in April 1994. An operative cholangiogram was performed which showed a large stone at the distal end of the common bile duct. This was removed laparoscopically. The upper and lower ends of the bile duct was explored with a choledochoscope and reported to be free of stones though it was noted that visualisation was difficult. A T-tube was positioned within the CBD.

The patient presented 8 months later with a 6 × 4 cm firm mass, superficial to the right hip joint deeply fixed to the underlying tissues anorexia and a 12 kg weight loss. A CT scan showed a mass with a cystic centre and thickened periphery. Ultrasound revealed echogenic shadows of uncertain aetiology.

The mass was excised and an abscess cavity containing two large faceted gallstones and several small fragments of stones was found. No connection to the peritoneal cavity was present. The abscess extended

*Correspondence to*: Peter T. Chin: 20/454 Edgecliff Rd, Edgecliff, New South Wales. 2027. Australia.

over the right hip joint. The cavity was drained and uneventful recovery followed. Culture of the pus grew mixed faecal flora. Her subsequent health has been good and weight gain recorded.

### CASE 2

A 77 year old woman underwent laparoscopic cholecystectomy in July 1994 with extraction of the gallbladder via the epigastric port. A retrieval device was not used. She presented over the ensuing 2 months with increasing pain and a tender mass in the left hypochondrium. A CT scan revealed a 3 × 2cm dense ovoid calcified mass in the left hypochondrium. A laparoscopy was performed with drainage of an intraperitoneal abscess and removal of a pigmented calculus. She made an uneventful recovery.

### CASE 3

An 80 year old woman with symptomatic cholelithiasis, underwent laparoscopic cholecystectomy in May 1994. She developed vague abdominal discomfort over the following 5 months and presented in October 1994 with a persistently discharging sinus. Exploration of the sinus revealed a pigmented gallstone in the right suprahepatic space. The sinus was laid open and healing by secondary intention was successful.

### METHOD AND RESULTS

A search of the current literature was undertaken utilising the "Medline" facility. Keywords used included: Retained, Spilled, Gallstones, Complications, laparoscopic cholecystectomy and abscess. All reports pertaining to complications of retained gallstones were included. The types of complications encountered and "time to presentation" were analysed and are presented below.

Case 1-Delayed "dumbell" shaped abscess with one component intraabdominal and the other subcutaneous. Both components contained gallstones[16]. *E. Coli* cultured. (28 weeks)

Case 2-Persisting discharging abdominal wall sinus. Gallstones in abdominal wall[10]. (24 weeks)

Case 3-Liver abscess containing stones[11]. (36 weeks)

Case 4-Subhepatic inflammatory mass[12]. Cultures negative for growth. (8 weeks)

Case 5-Intraabdominal abscess formation[14]. *E. Coli* cultured. (20 weeks)

Case 6-Microabscess and granuloma formation[19]. *Klebsiellla pneumonia* cultured. (20 weeks)

Case 7-Peritoneal-Cutaneous sinus tract formation[25]. *E. Coli* cultured. (32 weeks)

Case 8-Peritoneal-Cutaneous sinus tract formation[24]. (60 weeks)

Case 9-"Gallstone hip". Mixed faecal flora cultured. (32 weeks)

Case 10-Peritoneal abscess formation. *E. Coli* cultured. (8 weeks)

Case 11-Peritoneal-Cutaneous sinus tract formation. (11 weeks)

Average time to presentation 27.3 weeks.

### DISCUSSION

Stone spillage generally occurs during gallbladder manipulation causing perforation, or during the extraction phase of the operation. Stones may be retained in the peritoneal cavity or in the abdominal wall, as a potential source of infection. Spilled pigment stones, spillage from an acutely inflamed gallbladder or large volume gallstone spillage are suggested to be of particular concern[3]. Calcium Bilirubinate and pigment stones have been shown to contain bacteria[28,23].

What is the fate of the retained gallstone? This question has led to several experimental studies in an attempt to find the answer. Mixed sterile gallstones were collected and placed into the peritoneal cavity of 10 mongrel dogs. The stones were placed at the right subphrenic space, subhepatic area and in an omental "pocket". This experiment showed that there is no strong evidence of stone absorption, and calculi lost near the subphrenic space can erode the liver's convex surface, and there is then the potential for vessels of bile ducts to be injured[15]. Increased adhesions and abscess formation has been shown in the rat model[26,21,27]. Gallstones transplanted into the peritoneal cavity of rabbits revealed localised fibrosis with foci of fat necrosis. As part of the same study, 11 patients with retained gallstones were followed with one case of omphalitis reported[29].

Soper *et al.* reported a series of 250 laparoscopic cholecystectomies in which gallstone spillage due to gallbladder perforation occured in 32% of cases[17]. There were no intraabdominal infection, and no difference in wound infections between those patients with or without gallbladder perforations.

With improved technique and instruments, perforations of the gallbladder are becoming less frequent[2]. Retrieval devices such as the Pleatman Sac™ (Cabot Medical, Langhorne, PA), Endopouch™ (Ethicon Inc. Cincinatti, OH), and Lapsac™ (Cook Surgical, Bloomington, IN) were introduced to facilitate the extraction phase of the procedure. When a gallbladder is markedly inflamed and distended, insertion of such devices may minimise the difficulty in removal of the organ[3] and prevent gallstones being lost in the abdominal wall during the extraction phase.

Case 1 is an unusual case and is discussed separately. Gallstone spillage was thought to have occurred during the laparoscopic procedure, resulting in stones remaining in the right paracolic gutter postoperatively. This occurred during laparoscopic exploration and removal of common duct stones, where the extracted stones are placed on the omentum while further exploration is undertaken. A chronic abscess subsequently developed which migrated to the subcutaneous tissue, along the pressure gradient through the path of least resistance, the lumbar triangle. It is interesting to note that her postoperative symptoms did not include fever and she did not undergo prolonged antibiotic therapy. The use of a retrieval device to place extracted stones in, may have avoided this.

The rate of gallstone spillage has proved difficult to establish as it is not well documented. A spillage rate between 13% and 40%[2,4,17] has been reported and authors note thatfine sediment and gallstones that disappear into the posterior regions of the abdominal cavity are not always retrievable. A review of several large studies has failed to find rates of gallstone spillage[5–9].

Complications related to gallstone spillage are appearing in the literature and include; Peritoneal – Cutaneous Sinus tracts[10,16,20,24,25], Intraabdominal abscess[14], Subhepatic inflammaotry mass [12], Persistent discharging trocar sites[3], Liver abscess[11], Micro abscesses and granuloma formation[19], Retroperi toneal abscess formation and Cholelithoptysis[18]. Formation of an isolated subcutaneous abscess following laparoscopic cholecystectomy and stone migration has not yet been described.

### CONCLUSION

Spilled gallstones during laparoscopic cholecystectomy were initially thought to run a benign course with several studies supporting this view [17,29,30]. Recent reports highlight other possible outcomes of spilled stones. There is a paucity of data on the rate of spillage, as this event is rarely documented. An objective analysis of the rates of complications due to gallstone spillage is currently impossible.

We recommend the use of good quality instruments during manipulation of the gallbladder to minimise the risk of perforation and the use of retrieval devices to facilitate safe extraction of the gallbladder. Documentation of gallstone spillage is advised. Awareness of the delayed presentation (average 27 weeks) of complicated retained stones will assist in the early diagnosis of such events. All spilled gallstones should be removed. Appropriate evidence on the zeal with which one must pursue gallstone spillage is lacking. A prospective study on the type, rate, effort to retrieve, volume and outcomes of spilled gallstones in underway to fill this gap.

### REFERENCES

1. Soper, N.J., Stockmann, P.T., Dunnegan, D.L. and Ashley, S.W. (1992) Laparoscopic Cholecystectomy: The "New Gold standard". *Arch. Surg.*, **127**, 917
2. Donohue, J.H., Farnell, M.B., Grant, C.S., Von Heerdan, J.A., Wahlstrom, H.E., Sarr, M.G., Weaver, A.L. and Ilstrup, D.M. (1992) Laparoscopic Cholecystectomy: Early Mayo Experience *Mayo Clin. Proc.*, **67**, 449–455.
3. Cacdac, R.G., Lakra, Y.P: Abdominal wall sinus tract secondary to gallstones (1993). A complication of laparoscopic cholecystectomy. *Jour. Lap. Surg.*, **3(5)**, 509–511
4. Peters, J.H., Gibbons, G.D. and Innes, J.T., et al (1991) Complications of laparoscopic cholecystectomy. *Surgery.*, **110** (4), 769–778.
5. The Southern Surgeons Club; A prospective analysis of 1518 Laparoscopic cholecystectomies. *New Eng. J of Med.*, **324**, 1073–1078.
6. Cushieri, A., Dubois, F., Mouiel, J., Mouret, P., Becker, H., Buess, G., Trede, M. and Troidl, H. (1991) The European experience with laparoscopic cholecytectomy. *Am. J. Surg.*, **161**, 385–387.
7. Berci, G., Sackier, J.M. (1991) The Los Angeles experience with laparoscopic cholecystectomy. *Am.J.Surg.*, **161**, 382–384.
8. Zucker, K.A., Bailey, R.W., Gadacz, T.R. and Imbembo, A.L. (1991) laparoscopic guided cholecystectomy. *Am. J. Surg.*, **161**, 36–42.
9. Ponsky, J.L. (1991). Complications of laparoscopic cholecystectomy *Am.J.Surg.*, **161**, 393–395.
10. Ligam, K., Carter, R. and Drury, J.K. (1993) Retained gallstones following cholecystectomy. *J.R. Coll.Surg.Edinb.*, **38**, 353.
11. Wilton, P.B., Andy, J.R.O.J., Peters, J.J., Thomas, C.F., Patel, V.S. and Scott-Conner, C.E.H. (1993) Laparoscopic cholecystectomy, Leave no (spilled) stone unturned. *Surg. Endosc.*, **7**, 537–538.
12. VanBrunt, P.H., Lanzafane, R.J. (1994) Subhepatic Inflammatory Mass after laparoscopic cholecystectomy. *Arch. Surg.*, **129**, 882–883.
13. Wolfe, B.M., Gardiner, B.N., Leary, B.F. and Frey CF (1991) Endoscopic cholecystectomy. An analysis of complications. *Arch. Surg.*, **126**, 1192–1198.

14. Tschmelitsch, H., Glasser, K., Klingler, P. and Bodrev, E. (1993) Late complication caused by stone spillage during Laparoscopic Cholecystectomy. *The Lancet*., **342**, 369.
15. Cohen, R.V., Periera, P.R.B., de Baros, M.V., Ferriera, E.A.B. and de Tolosa, E.M. (1994) Is retrieval of lost peritoneal gallstones worthwhile? *Surg Endosc.*, **8**, 1360
16. Mellinger, J.D., Eldridge, T.J., Eddelman, E.D. and Crabbe, M.M. (1994) Delayed gallstone abscess following laparoscopic cholecystectomy. *Surg Endosc.*, **8**, 1332–1334
17. Soper, D.J., Dunnegan, D.L. (1991) Does laparoscopic gallbladder perforation influence the early outcome of laparoscopic cholecystectomy? *Surg Laparosc Endosc.*, **1**, 156–161
18. Downie, G.H., Robbins, M.K., Souza, J.J. and Paradowski, L.J. (1993) Cholelithoptysis: a complication of laparoscopic cholecystectomy. *Chest.*, **103**, 616–617
19. Golub, R., Nwogu, C., Cantu, R. and Stein, H. (1994) Gallstone shrapnel contamination during laparsocopic cholecystectomy. *Surg. Laparosc. Endosc.*, **8**, 898–900
20. Catarci, M., Zaraca, F., Scaccia, M. and Carboni, M. (1993) Lost intraperitoneal stones after laparoscopic cholecystectomy: harmless sequelae or reason for reoperation? *Surg. Laparosc. Endosc.*, **3**, 318–322
21. Sax, H.C., Adams, J.T. (1993) The fate of the spilled gallstone. *Arch. Surg.*, **128**, 469
22. Jacob, H., Rupin, K.P., Cohen, M.C., Kalin, I.J. and Kan, P. (1979) Gallstones in a retroperitoneal abscess: a late complication of perforation of the gallbladder. *Dig. Dis. Sci.*, **24**, 964–996
23. Stewart, L., Smith, A.L., Pelligrini, C.A., Motson, R.W. and Way, L.W. (1987) Pigmented gallstones form as a composite of bacterial microcolonies and pigment solids. *Ann. Surg.*, **206**, 242–250
24. Stearman, P.H. (1994) Delayed peritoneal-cutaneous sinus from unretrieved gallstones. *Surg. Laparosc. Endosc.*, **4**, 452–453
25. Gallinero, R.N., Miller, F.B. (1994) The lost gallstone. Complication after laparoscopic cholecystectomy. *Surg. Laparosc. Endosc.*, **8**, 913–914
26. Johnston, S., O'Malley, K., McEntee, G., Grace, P., Smyth, E. and Bouchier-Hayes, D. (1994) The need to retrieve the dropped stone. *Am. J. Surg.*, **167**, 608–610
27. Leland, D.G., Dawson, D.L. (1993) Adhesions and experimental intraperitoneal gallstones. *Contemp. Surg.*, **42**, 273–275
28. Smith, A.L., Stewart, L., Fine, R., Pelligrini, C.A. and Way, L.W. (1989) Gallstone disease. The clinical manifestations of infectious stones. *Arch. Surg.*, **124**, 629–633
29. Welch, N., Hinder, R.A., Fitzgibbons, R.J. and Rouse, J.W. (1991) Gallstones in the peritoneal cavity: a clinical and experimental study. *Surg. Laparosc. Endosc.*, **1**, 246–247
30 Lee, V.S., Paulson, E.L., Libby, E., Flannery, S.E. and Meyers, W.C. (1993) Cholelithoptysis and Cholelithorrhea: Rare Complications of laparoscopic Cholecystectomy *Gastroentorology.*, **105**, 1877–1881

